# Improvement of image quality for small lesion sizes in ^18^F-FDG prone breast silicon photomultiplier-based PET/CT imaging

**DOI:** 10.22038/aojnmb.2024.78080.1553

**Published:** 2025

**Authors:** Nobuhiro Yada, Hiroyuki Kuroda, Toshihiko Kawamura, Mizuki Fukuda, Yoshinori Miyahara, Takeshi Yoshizako, Yasushi Kaji

**Affiliations:** 1 Department of Radiology, Shimane University Hospital, Izumo, Japan; 2 Department of Radiology, Faculty of Medicine, Shimane University, Izumo, Japan; 3 Division of Medical Informatics, Shimane University Hospital, Izumo, Japan

**Keywords:** Breast cancer, Digital PET, Prone position

## Abstract

**Objective(s):**

We investigated image quality and standardized uptake values (SUVs) for different lesion sizes using clinical data generated by ^18^F-FDG-prone breast silicon photomultiplier (SiPM)-based positron emission tomography/computed tomography (PET/CT).

**Methods:**

We evaluated the effect of point-spread function (PSF) modeling and Gaussian filtering (Gau) and determined the optimal reconstruction conditions. We compared the signal-to-noise ratio (SNR), contrast, %coefficient of variation (%CV), SUV, and Likert scale score between ordered-subset expectation maximization (OSEM) time-of-flight (TOF) and OSEM+TOF+PSF in phantom and clinical studies. The conventional image was generated with OSEM+TOF_Gau 6 mm. The National Electrical Manufacturers Association body phantom with 10-mm hot sphere data was acquired for 5 min. Twenty-six patients (40 lesions, ranging from 3.7 to 63.0 mm) were examined using prone breast PET/CT with a breast positioner for breast cancer staging. PET data were acquired 125±9.7 min after intravenous injection of 220±16.1 MBq at 5 min/bed.

**Results:**

In the phantom study, a high SNR was obtained from a 3- to 5-mm Gaussian filter for OSEM+TOF+PSF. The contrast obtained with OSEM+TOF without Gaussian filtering was superior to that obtained with OSEM+TOF+PSF_Gau 4 mm. In the clinical study, the image quality depended on lesion size. The average SNR was significantly higher at 40.8% for lesions >20 mm with OSEM+TOF_Gau 6 mm than with OSEM+TOF without Gaussian filtering. The average contrast for lesions ≤10 mm was significantly higher by 42.0% with OSEM+TOF without Gaussian filtering than with OSEM+TOF_Gau 6 mm. The average SUV_max_ of OSEM+TOF without Gaussian filtering significantly increased by 53.3% for lesions ≤10 mm.

**Conclusion:**

OSEM+TOF without Gaussian filtering provided good contrast and quantitative value for small lesions.

## Introduction

 In oncology, 2-^18^F-fluoro-2-deoxy-D-glucose (^18^F-FDG) positron emission tomography/ computed tomography (PET/CT) imaging is useful for initial and recurrence staging at the diagnosis of breast cancer (1). For breast cancer diagnosis, prone breast PET exhibited improved sensitivity and accuracy compared with supine whole-body PET (2). For clinical examination, prone breast PET data are generally acquired after supine whole-body PET imaging. On the other hand, the standardized uptake value (SUV) measurement was influenced by a partial-volume effect (PVE) (3). The maximum SUV (SUV_max_) of breast cancer lesions is low at sizes less than 2 cm (4), and Heinisch et al. (5) reported that PET struggled to achieve reliable imaging of carcinomas smaller than 10 mm. 

 Several clinical PET/CT systems with silicon photomultipliers (SiPM) have improved energy and time resolutions (6). The system performance of Vereos PET/CT based on the National Electrical Manufacturers Association (NEMA) NU2 tests were validated in a prior study. The PET/CT system improved the accuracy of time-of-flight (TOF) information. 

 The application of point-spread function (PSF) modeling has increased the signal intensity of lesions and thereby improved image quality, lesion detectability, and diagnostic confidence (7-11). Therefore, SiPM-based PET/CT systems may be beneficial for detecting small lesions. 

 The performance of SiPM-based PET/CT still needs to be clarified with clinical data. To our knowledge, no previous study has investigated the differences in image quality and lesion size using prone breast PET/CT imaging. The image quality depends on the lesion size with the combination of PSF modeling and Gaussian filtering. Thus, we investigated the image quality and SUV for different lesion sizes using prone breast SiPM-based PET/CT.

## Methods

 This retrospective study was performed in line with the principles of the Declaration of Helsinki. The Ethical Review Committee of our institution approved this study (Grant Number: 6004) and the requirement to obtain informed consent was waived.

***Phantom data***

 We used a NEMA body phantom (Pro-NM NEMA NU2, Pro-Project, Okszów, Poland) image of a 10-mm sphere with 4:1 background (BG) activity of 1.5 kBq/mL ^18^F-FDG that was adjusted to the radioactivity concentration of the mammary gland. The data were acquired for 5 min and reconstructed with OSEM+TOF and OSEM+TOF+PSF with 4 iterations and 10 subsets with and without Gaussian filtering. We previously determined the optimal number of iterations using the phantom image acquired for 100 min.

***Patient data***

 Twenty-six female patients (Table 1) were examined using breast ^18^F-FDG-PET/CT with a magnetic resonance image mammography support device (Philips Healthcare, Orange, OH) for breast cancer staging. All patients’ lesions were found on biopsy to be invasive ductal carcinoma. The correlations between the histological and breast imaging findings were examined by radiologists and pathologists. PET data were acquired for 5 min/bed after supine whole-body imaging. All patients had fasted for >6 h.

**Table 1:** Characteristics of 26 patients with invasive ductal carcinoma

**Patient characteristics**	**Mean±SD, range**
Age (years)	60.0±11.3 (40-79)
Body weight (kg)	60.0±8.8 (43.1-80.5)
Blood glucose (mg/dL)	107±14.6 (89-144)
Dose (MBq)	220±16.1 (187-251)
Time between injection and scan (min)	125±9.7 (111-144)
**Lesion N, size (mm)**	
Lesions ≤10 mm	15, 7.1±1.7 (3.7-9.6)
10 mm < lesions ≤20 mm	15, 14.3±2.2 (11.0-19.0)
Lesions >20 mm	10, 32.1±14.5 (20.4-63.0)

***Image acquisition and processing***

 PET data were acquired in 3D list-mode using a SiPM-based PET/CT system (Vereos PET/CT, multi-slice CT scanner, Philips Healthcare) (7, 8). The data were reconstructed using the 3D ordered-subset expectation maximization (OSEM; iterations, 4; subsets, 10)+TOF and OSEM+TOF+PSF modeling (PSF, 1; iterations, 1; regularization, 6) (7) with and without Gaussian filtering. The voxel size was 2 mm. The CT data were acquired for attenuation correction under the following conditions: tube voltage, 120 kV; absolute minimum tube-current time product, 30 mAs; iDose4, level 4.

***Evaluation***

***Phantom study***

 We calculated the signal-to-noise ratio (SNR), contrast, and %coefficient of variation (%CV) as follows:

$\mathrm{SNR}=\frac{C_{10\mathrm{mm}}-C_{\mathrm{BG}}}{\sigma_{\mathrm{BG}}}$

$\mathrm{Contrast}=\frac{C_{10\mathrm{mm}}}{C_{\mathrm{BG}}}$

$\mathrm{\%CV}=\frac{\sigma_{\mathrm{BG}}}{C_{\mathrm{BG}}}\times 100(\%)$

 Here, C_BG_ is the mean number of counts for the BG region of interest (ROI) with a diameter of 10 mm, σ_BG_ is the mean of the standard deviation (SD) of the BG ROI; and C_10 mm_ is the maximum number of counts for the 10-mm diameter ROI in the central slice of the 10-mm sphere. The BG ROI (n=60) covered five slices, including the central slice of the 10-mm sphere.

 Gaussian filtering was used for post-filtering (full width at half-maximum: FWHM=0, 1, 2, 3, 4, 5, 6, 7, 8, 9, 10, 11, and 12 mm).

***Clinical study***

 We evaluated the SNR, lesion contrast, %CV of the mammary gland, SUV_max_, and the Likert scale score. Breast cancer lesions were divided into three groups as follows: ≤10 mm, >10 mm to ≤20 mm, and >20 mm. The FWHM Gaussian conditions were without filtering and 4 mm, which was twice that of the voxel. We assessed the effect of the PSF modeling and Gaussian filtering in the OSEM+TOF, OSEM+TOF with a 4-mm FWHM Gaussian filter (OSEM+TOF_Gau 4 mm), and OSEM+TOF+PSF with a 4-mm FWHM Gaussian filter (OSEM+TOF+PSF_Gau 4mm). To clarify the superiority of the novel PET/CT image, the image quality was additionally compared with that of conventional images that were obtained with a recovery coefficient (RC) within the maximal and minimal RC in the European Association of Nuclear Medicine procedure guidelines for ^18^F-FDG-PET/CT imaging (12,13) as follows: OSEM+TOF reconstruction (4 iterations and 10 subsets). 

 The FWHM of the Gaussian filter was set at 6 mm (OSEM+TOF_Gau 6 mm). The ROI within the mammary gland was placed on the healthy side. The ROI of the breast cancer lesion was placed on its maximum diameter. The size of each lesion was manually measured on the CT image.

 In addition, two readers, radiologist and radiological technologist who had 20 and 13 years of experience in nuclear medicine, evaluated the PET/CT images according to a 5-point Likert scale score (1: definitely negative, 2: probably negative, 3: indeterminate, 4: probably possible, 5: definitely possible).

***Data analysis***

 Data were analyzed using π.pmod software (PMOD Technologies LLC, Zurich, Switzerland) and JMP Pro 16.1.0 (SAS Institute Inc., Cary, NC). 

 The SNR and contrast were then calculated with the exclusion of mammary glands with an SUV<1.0. The inter-reader agreement was analyzed using the kappa statistics (14). The Likert scale score was then divided into two groups as follows: possible denoted by 4 and 5, and negative denoted by 1-3. The results are expressed as the mean and SD. All data were analyzed using a paired t-test (p<0.05).

## Results

***Phantom study***

**Figure 1:**
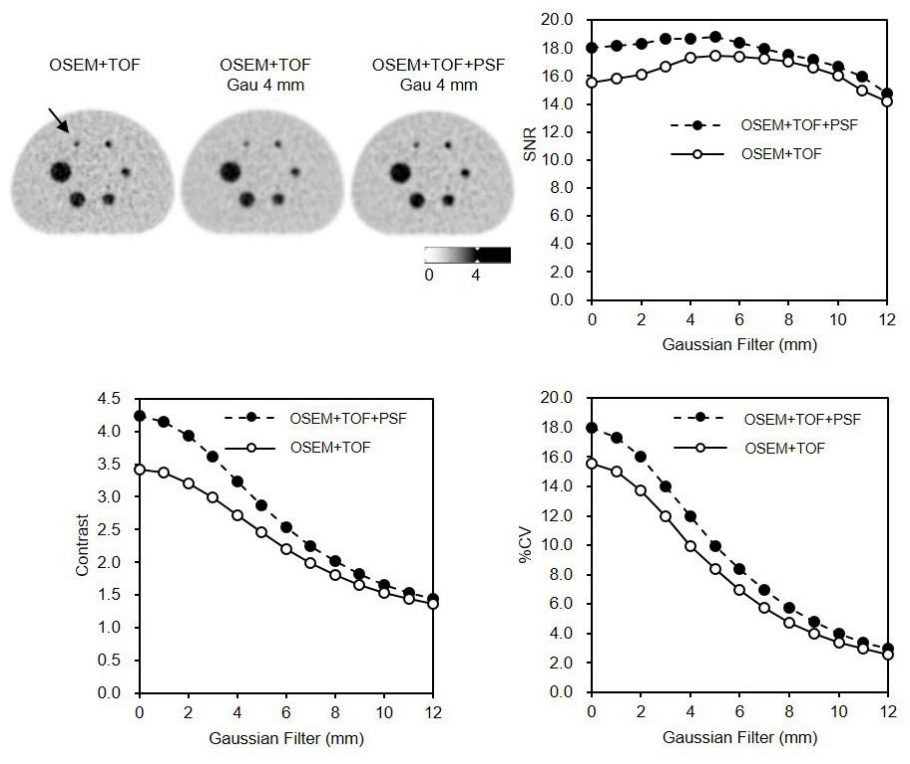
PET/CT images reconstructed by OSEM+TOF, OSEM+TOF with 4-mm Gaussian filter and OSEM+TOF+PSF with 4-mm Gaussian filter. SNR, contrast, and %CV were evaluated at various FWHMs of the Gaussian filter on the 10-mm hot sphere (**arrow**)

 In the phantom study, a high SNR was obtained from a 3- to 5-mm Gaussian filter for OSEM+TOF+PSF. The SNR was 18.7, 17.3, and 15.6 for OSEM+TOF+PSF_Gau 4 mm, OSEM+TOF_Gau 4mm, and OSEM+TOF, respectively (Figure 1). The contrast was 3.42, 3.24, and 2.73 for OSEM+TOF, OSEM+ TOF+PSF_Gau 4 mm, and OSEM+ TOF_Gau 4 mm, respectively. The contrast obtained with OSEM+TOF was superior to that obtained with OSEM+TOF+PSF_Gau 4 mm. The %CV was 10.0, 12.0, and 15.5 for OSEM+TOF_Gau 4 mm, OSEM+TOF+PSF_Gau 4 mm, and OSEM+TOF, respectively.

***Clinical study***

**Figure 2:**
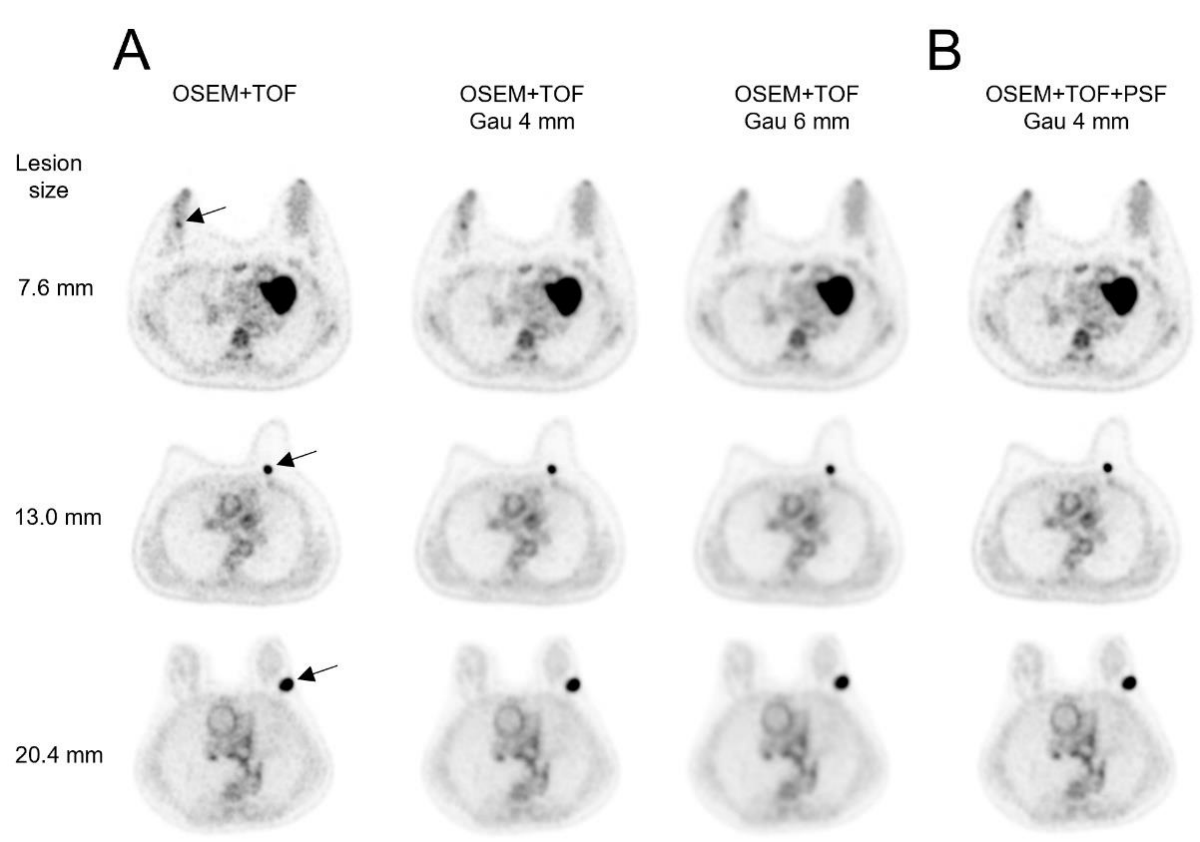
Clinical PET/CT images of 3 patients with lesions of different sizes (**arrow**). SUV_max_ in OSEM+TOF: upper, 3.1; middle, 9.1; bottom, 6.7

**Figure 3:**
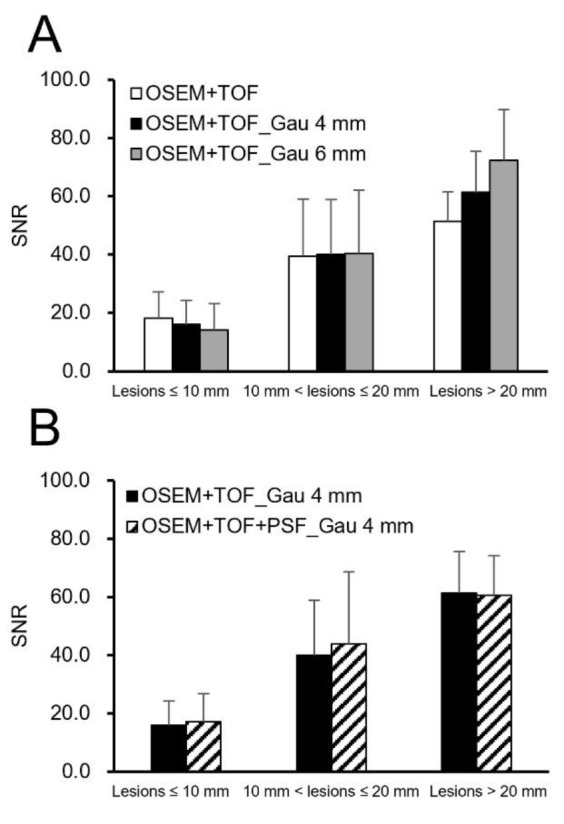
Signal-to-noise ratios among different reconstruction conditions (**A:** Gaussian filter, **B:** PSF modeling) for different lesion sizes

**Figure 4:**
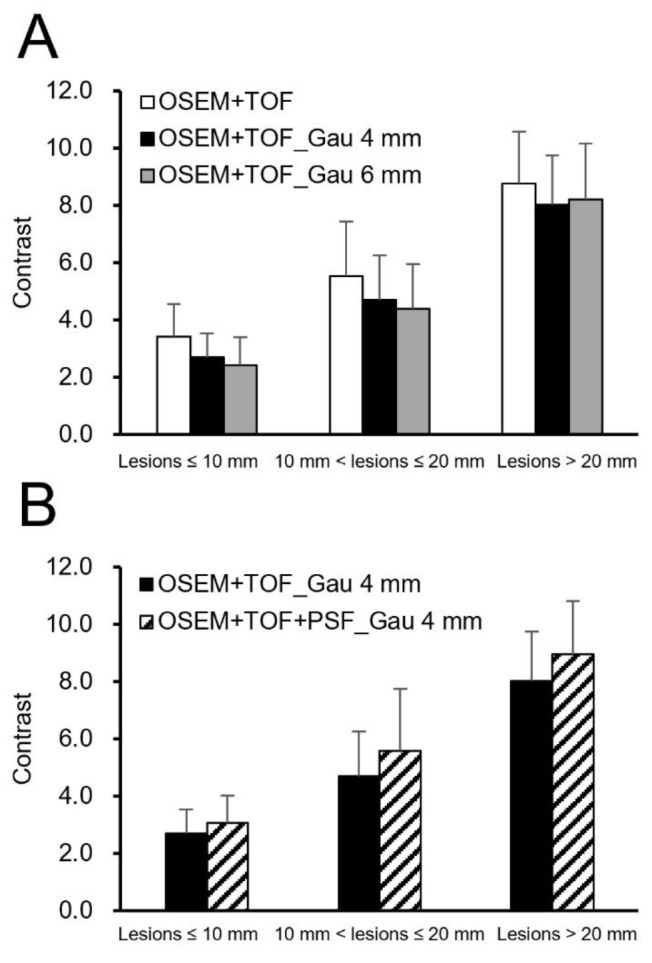
Contrast values among different reconstruction conditions (**A**: Gaussian filter, **B**: PSF modeling) for different lesion sizes

**Figure 5:**
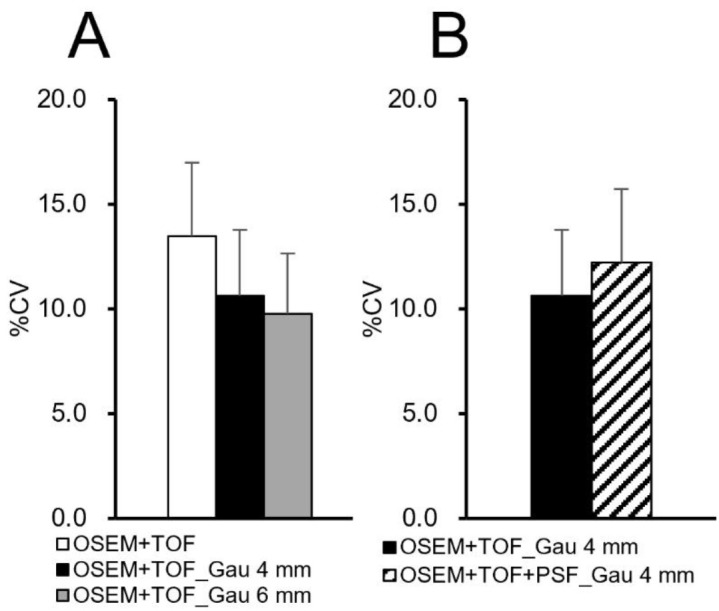
Relationship between the uniformity of the mammary gland on the healthy side and the reconstruction conditions (**A**: Gaussian filter, **B**: PSF modeling)

**Figure 6:**
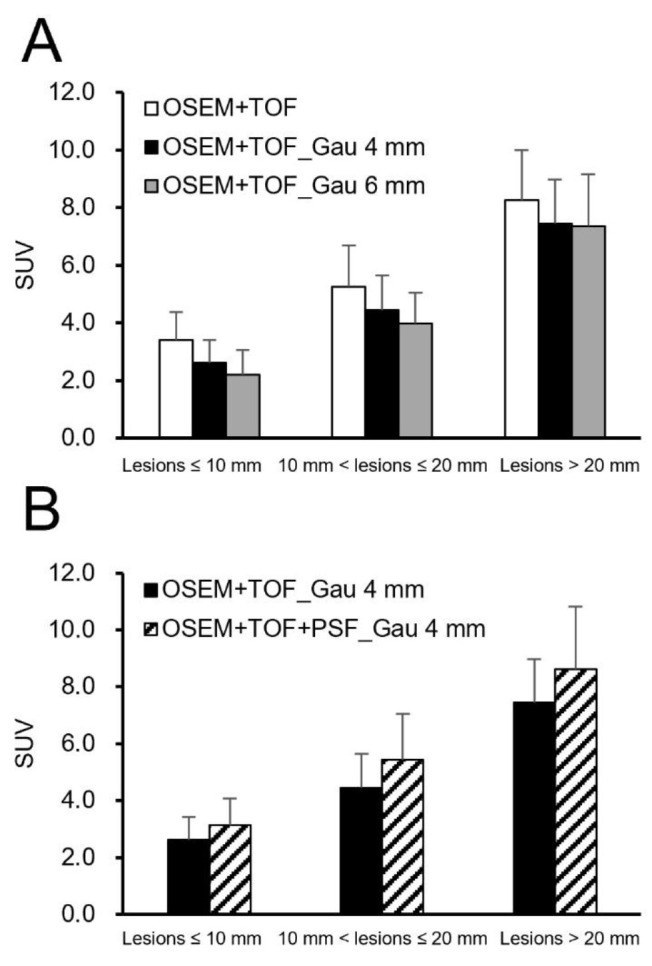
Distribution of maximum standardized uptake values for different lesion sizes

**Figure 7:**
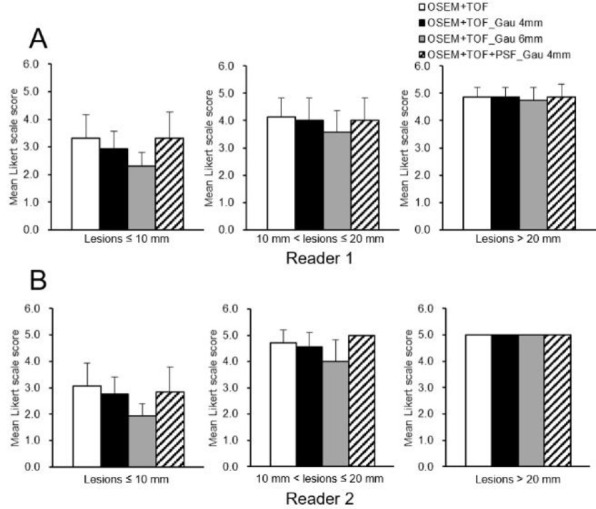
Mean Likert scale score among different reconstruction conditions (a reader 1, b reader 2) for different lesion sizes

Figure 2 shows the clinical PET/CT images generated from 3 patients. Both small and large lesions were detected. The image quality depended on lesion size, particularly for lesions ≤10 mm. The average SNR was significantly higher at 40.8% for lesions >20 mm with OSEM+TOF_Gau 6 mm than with OSEM+TOF (Figure 3, Table 2). For lesions ≤10 mm, the average contrast was significantly higher by 42.0% with OSEM+TOF than with OSEM+TOF_Gau 6 mm (Figure 4). The average %CV significantly decreased by 21.1% with OSEM+TOF_Gau 6 mm compared with OSEM+TOF (Figure 5). In most cases, good SNR, contrast, and %CV were provided with OSEM+TOF and OSEM+TOF_Gau 6 mm. The average SUV_max_ of OSEM+TOF significantly increased by 53.3% for lesions ≤10 mm and by 31.8% for lesions >10 mm to ≤20 mm compared with OSEM+TOF_Gau 6 mm (Figure 6). The average Likert scale score of OSEM+TOF was significantly increased at 1.0 (Reader 1) and 1.2 (Reader 2) for lesions ≤10 mm compared with OSEM+TOF_Gau 6 mm, respectively (Figure 7, Table 3). The inter-reader agreement was substantial (kappa=0.622, p<0.0001).

**Table 2:** Comparison of image quality and maximum standardized uptake values differences between OSEM+TOF and OSEM+TOF_Gau 6 mm

	**SNR**	**Contrast**	**%CV**	**SUV**
Lesions ≤10 mm	n.s.	p<0.05	-	p<0.05
10 mm < lesions ≤20 mm	n.s.	n.s.	-	p<0.05
Lesions >20 mm	p<0.05	n.s.	-	n.s.
Mammary gland	-	-	p<0.05	-

**Table 3:** Comparison of Likert scale scores between OSEM+TOF and OSEM+TOF_Gau 6 mm

	**Reader 1**	**Reader 2**
Lesions ≤10 mm	<0.05	p<0.05
10 mm < lesions ≤20 mm	n.s.	n.s.
Lesions >20 mm	n.s.	n.s.

## Discussion

 High SNR was obtained from the PET image with the theoretically optimal Gaussian filter FWHM of 4 mm using PSF modeling (Figure 1). 

 For the Vereos PET/CT system, the OSEM+TOF PET reconstruction was applied with a <4-mm FWHM Gaussian filter (11). 

 According to the NEMA NU2-2012 standard, the contrast recovery coefficient (CRC) for a 10-mm sphere is 54.4% (7). By applying PSF, 62% contrast recovery is achieved (8). PET reconstruction with PSF and a smaller voxel size gives a high RC for small hot spheres (11). Image quality generally has a trade-off between contrast and %CV. The Gaussian filter improved the uniformity and then smoothed the distributions of the signal. On the other hand, the %CV was lower using PSF modeling for other PET system (15). The optimal reconstruction conditions are often evaluated with the CRC, SNR, contrast, and %CV because the PET image varies with the combination of TOF, PSF, and the FWHM of the Gaussian filter. 

 The contrast was superior for OSEM+TOF than for OSEM+TOF+PSF_Gau 4 mm. Our findings suggest that the optimal FWHM Gaussian conditions are without filtering and 4 mm. 

 Moreover, filtering is not required to detect small lesions. Low spatial resolution is often a problem in nuclear medicine imaging, resulting in decreased detection of small lesions, and PET/CT is no exception.

 For lesions >20 mm, a higher SNR was obtained for OSEM+TOF_Gau 6 mm than for OSEM+TOF (Figure 3). The SNR and contrast depended on the lesion size due to the difference in the PVE. For lesions ≤10 mm, the contrast was high with OSEM+TOF (Figure 4). 

 Thus, good SNR, contrast, and %CV was not obtained overall with OSEM+TOF_Gau 4 mm and OSEM+TOF+PSF_Gau 4 mm. Therefore, we did not need to compare the results of OSEM+TOF_Gau 4 mm and OSEM+TOF+ PSF_Gau 4 mm with that of other conditions. 

 Compared with OSEM+TOF_Gau 6 mm, the SUV_max_ of OSEM+TOF significantly increased as the lesion size decreased (Figure 6). A high CRC is obtained with PET/CT images of hot spheres greater than 22 mm (7, 8). For lesions ≤20 mm, the SUV_max_ with OSEM+TOF_Gau 6 mm was greatly decreased because the PET/CT image showed deteriorated spatial resolution with both low CRC and smoothing. The SUV with PSF modeling was not used in the clinical study because the effect of PSF modeling depends on lesion size with Gibb’s oscillation (16), and the difference in the RC (i.e., the SUV is not linearly related to the count). We do not recommend that Gaussian filtering be used with prone breast PET/CT if a high spatial resolution is being used. The PET/CT image without gaussian filtering had higher average Likert scale scores because of higher SNR and contrast. 

 The prone PET/CT imaging with 2-mm voxel image was applied to detect the primary legions (17). Furthermore, the PET images with improved the contrast is needed to detect the small primary legions. Compared with OSEM+TOF_Gau 6 mm, the SUV_max_ and average Likert scale score increased for OSEM+TOF, and the SNR and %CV was thus inferior. 

 Importantly, underestimation of the SUV may lead to a serious error in clinical practice (18). 

 In addition, the spatial resolution of the off-center position in our PET/CT system was superior to that of not only a conventional PET/CT system (19), but also another SiPM-based PET/CT system (7).

## Conclusion

 OSEM+TOF reconstruction with a 2-mm voxel and without filtering achieves good contrast and quantitative value for small lesions.
